# Novel fabrication of soft microactuators with morphological computing using soft lithography

**DOI:** 10.1038/s41378-019-0092-z

**Published:** 2019-09-23

**Authors:** Manav Tyagi, Jingle Pan, Edwin W. H. Jager

**Affiliations:** Sensor and Actuator Systems, Department of Physics, Chemistry, and Biology (IFM), Linköping University, Linköping, 58183 Sweden

**Keywords:** Electronic devices, Electrical and electronic engineering

## Abstract

A simple and cost-effective method for the patterning and fabrication of soft polymer microactuators integrated with morphological computation is presented. The microactuators combine conducting polymers to provide the actuation, with spatially designed structures for a morphologically controlled, user-defined actuation. Soft lithography is employed to pattern and fabricate polydimethylsiloxane layers with geometrical pattern, for use as a construction element in the microactuators. These microactuators could obtain multiple bending motions from a single fabrication process depending on the morphological pattern defined in the final step. Instead of fabricating via conventional photolithography route, which involves multiple steps with different chromium photomasks, this new method uses only one single design template to produce geometrically patterned layers, which are then specifically cut to obtain multiple device designs. The desired design of the actuator is decided in the final step of fabrication. The resulting microactuators generate motions such as a spiral, screw, and tube, using a single design template.

## Microrobots: Novel fabrication of artificial muscle

A novel technique can fabricate multiple ‘soft microrobots’ from a single design process that may prove useful in biomedical applications. With a continuous drive for robots to get smaller and more efficient, researchers are looking for alternatives to complex, traditional machinery. Edwin Jager and his team from Linköping University in Sweden now report a technique that combines elastomeric silicone and electroactive polymer into an artificial muscle: a soft microrobot actuated depending on the presence of ions in solution. Through controlling the thickness and length of rigid and flexible sections, the team’s devices can produce multiple, different motions such as spiraling and screwing. Such soft robots could find uses in future minimally invasive surgery or drug delivery systems. Jager and his team are now working on soft robots that function in air.

## Introduction

Microrobots are being developed for applications and tasks in the sub-millimeter domain, such as manipulation of small, biological objects and microsurgery. A large focus in microrobotics is dedicated towards decreasing the size of robots while enhancing the performance. Although miniaturization is highly desirable, shrinking conventional robots with their discrete actuation, control, and moving parts is challenging and too complex to realize. Recent work, where researchers developed molecular robots using natural proteins as actuator and control system^1^, demonstrates that developing actuators with multiple embedded properties provides a feasible approach to miniaturize robots while retaining their complex and efficient functionality.

Soft actuators or artificial muscles have been contributing to the progress of robotics, allowing for the fabrication of scalable and flexible robotic systems^2–5^. Soft robots, made up of compliant materials like artificial muscles, extend the adaptability in applications requiring flexibility, which is rather constrained by the rigidness of traditional robots. Soft robots, capable of mimicking the motion of living organisms, have been studied to demonstrate their locomotion^6,7^. Soft multilegged robots, mimicking octopus, can crawl and grasp fragile objects^8,9^. Since soft robotic systems are compliant, they are interesting for biomedical applications^10,11^, including applications towards minimal invasive surgery^12,13^ and drug delivery systems^14,15^.

Soft robotic systems have been driven by various actuation mechanisms^16,17^, such as based on variable length tendons^18^, shape memory alloys^19^, pneumatic actuator muscles (PAM)^20–22^, and flexible elastomeric actuators (FEAs), operated either pneumatically^23,24^ or hydraulically^25–27^. Presently, PAMs or FEAs are the prevalent technologies driving the soft robotic actuators at centimeter or larger scales. At the microscale, soft microrobots are operated by actuators based on electroactive polymers (EAPs), broadly classified under two categories^28,29^: electronic EAP, which actuate at high electric fields, and ionic EAP, which actuate due to the insertion/withdrawal of ions upon application of a low potential^30,31^.

Ionic EAPs, such as conducting polymers, are particularly interesting due to their attractive physical and electrical properties, such as high flexibility, high power-to-weight ratio, and low operating potential^10,32^. The volume change in conducting polymers such as polypyrrole (PPy) is based on the switching between different redox states under an applied potential, which results in an ion flow to and from the solution. Ions from the solution move inside the PPy matrix and to accommodate for the extra ions, the PPy swells. Reversing the potential causes ions to move out again^33^. Conducting polymer actuators have been microfabricated^34–36^ and used as microrobotic devices with different functionalities such as gripper or manipulator^3^, underwater flipper^37^, and even a micro-aerial vehicle^38,39^. Techniques used to pattern and fabricate polymer microactuators and soft microrobots are limited because of the compatibility of polymers with processing methods^17^, and include techniques such as photolithography^40^ and laser ablation^41,42^. Conventional microfabrication requires multiple chromium photomasks, as well as multiple photolithography steps on a silicon wafer^43^. Although offering precise definition and patterning, it is time consuming and requires harsh physical or chemical agents, complicating the manufacturing process. Keeping in mind the shortcomings of the conventional technology, we have developed a new process using soft lithography to pattern and fabricate conducting polymer microactuators. Soft lithography offers numerous advantages over the conventional ways to simplify the fabrication of polymer actuators.

Control of the motion of actuators often requires precise position sensing and feedback, for example, through integrated sensors, which adds to the complexity of device^44^. Consider the mammalian muscle movement, for example, where the bone–joint structure of the body helps to direct the motion of the limb. Such a supporting structure is absent in a soft microactuator. Therefore, integrating a control structure in the body of microactuator, for example, with geometrical patterns, will enable controlled movement utilizing morphological computation and would also simplify the process of scaling down the robots. Morphological computing exploits such passive construction patterns embedded in the actuator to enable a desired control over the motion^45,46^.

**Fig. 1:**
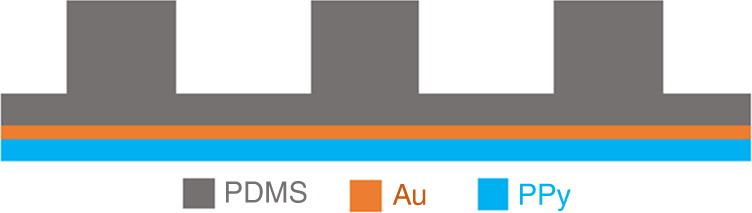
Cross-section of the unimorph actuator device consisting of a patterned PDMS layer, gold, and polypyrrole

**Fig. 2:**
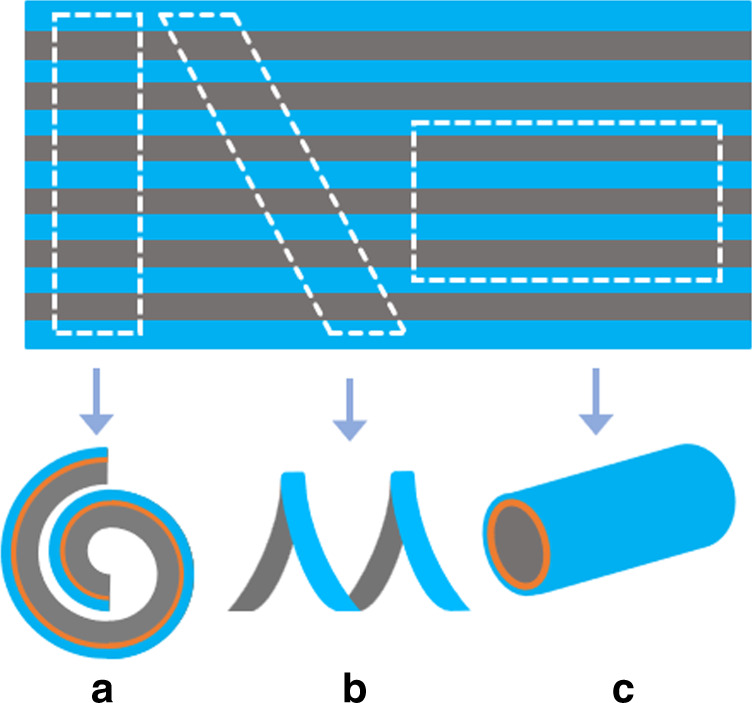
Control of the bending direction and shape. Top view of the device structure and indication of the different “cuts“ with respect to the patterned PDMS. The resulting movement of the actuator due to the “cut” with respect to the pattern is shown under each as spiral (**a**), screw (**b**), and tube (**c**)

To demonstrate morphological computing in soft microactuators, we designed a three-layer unimorph artificial muscle^47^ (Fig. 1), comprising of a polydimethylsiloxane (PDMS) layer patterned with geometrical design, a thin layer of gold (Au) and a thick layer of PPy. We employed soft lithography to fabricate patterned PDMS layers for use as passive construction element in actuator devices. This replication method allows for fabricating multiple PDMS layers from a single design template and enables a simpler fabrication process. To achieve controlled bending, the PDMS membrane was patterned with thin and thick sections of defined dimensions (Fig. 2). The thinner parts of PDMS membrane act as joints allowing for the bending of the microactuator, while the thicker parts, being too mechanically stiff to bend, act as rigid elements. For the purpose of simplicity, we call these thin and thick regions in the PDMS membrane as flexible and rigid sections, respectively.

**Fig. 3:**
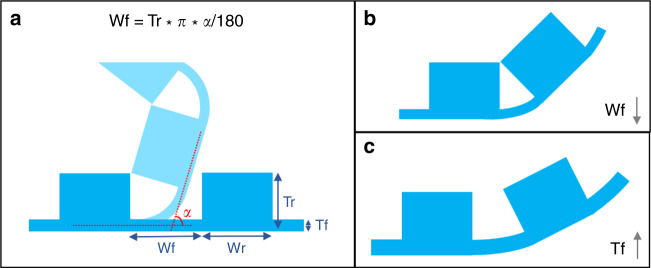
Control of the bending angle. **a** Dependence of bending angle on criticial morphological parameters of the actuator membrane, where Wf, Wr represents the width of flexible and rigid sections, and Tf, Tr the thickness of flexible and rigid sections respectively. **b** Dependence of bending angle on the width of flexible section; the bending angle lowers with the reduction of Wf. **c** Dependence of bending angle on the thickness of flexible section, the bending is not locked due to the increase in Tf resulting in lowering of bending angle

Morphological computation enables the control of the bending motion in two ways, first by controlling the bending direction, which defines the actuator shape (Fig. 2), and second by controlling the bending angle, which defines the curvature (Fig. 3). For the first concept, the patterned PDMS layer with PPy can be cut in various shapes after fabrication, to obtain multiple microactuators with different bending shapes without the need for several photomasks to define each step. The PDMS-Au-PPy layered structure, embedded with the geometrical design, allows for deciding on the type of actuator in the last stage of the fabrication process. That is, in the final step, the user can define the morphological computing pattern and cut the shape of the final actuator device depending on the kind of bending direction needed, for example, spiral, screw, or tube, independent of previous fabrication steps (Fig. 2). For the second, the PDMS pattern itself also determines the bending angle and thus the radius of curvature based on the geometrical patterns (Fig. 3a). By properly designing the thickness and length of the flexible (Tf, Wf) and rigid (Tr, Wr) sections, a predetermined maximum curvature can be achieved by locking the two neighboring rigid segments in place. For instance, a shorter length of the flexible part (decreased Wf) will lock the actuator in a larger curvature (Fig. 3b). This concept does require that the inherent bending angle of the flexible section is larger than the intended, locked bending angle. If, for instance, the inherent bending angle of the flexible segment is smaller, for example, due to a thicker flexible segment (Tf), then the rigid segments cannot lock in place and the actuator curvature is determined by the curvature of the flexible segment (Fig. 3c).

## Results and discussion

### Design and fabrication of unimorph microactuator

The bending direction and the bending angle of the microactuator depends on several factors, such as the volume change, thickness of each layer, the Young’s modulus, and the Poisson’s ratio of each layer within the actuator, and it can be modeled in its simplest form using the bending beam theory^48,49^. Assuming the Young’s modulus and Poisson’s ratio being constant for each layer in the actuator^48,50^ (PPy, Au, and PDMS in this case), the thickness (*t*) of each layer plays a vital role in determining the deflection (*d*) during actuation through the moment of inertia *I* of a beam with width *w*, thickness *t*, and Young’s modulus *E* (Eq. 1):

1 $$1/d\sim E \times I = E \times wt^3/12.$$

**Fig. 4:**
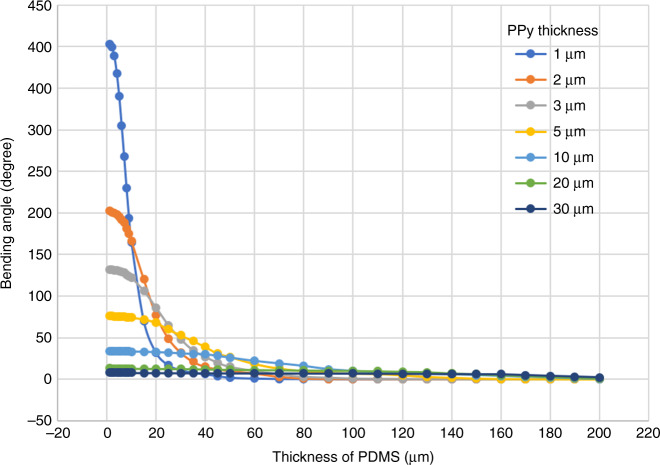
Simulation result of the bending angle of a tri-layer unimorph actuator (PPy-Au-PDMS) as a function of PDMS thickness for various polypyrrole thicknesses. The bending angle greater than 360° corresponds to curling of the actuator

This relationship was used to determine the proper dimensions of the flexible and rigid sections of PDMS, and the bending angles of the unimorph actuator were simulated based on PPy, Au and PDMS thicknesses, in accordance with the model parameters listed in Table 1^51–54^. The gold layer being very thin (~75 nm) compared to PDMS and PPy, has a minimal effect on the bending angle of the actuator. For a simplistic approach, we consider that each actuator layer is elastic and isotropic, and there is no mechanical deformation or delamination between the layers while bending. Figure 4 shows the variation of the bending angle with respect to the thickness of PDMS and PPy layers calculated using the bending beam theory, with the help of a simulation program^49^. As can be observed, the bending angle is nearly negligible with PDMS layers over 140 µm for the PPy thickness up to 30 µm. As the PDMS thickness reduces below 50 µm, the thickness of active material PPy starts to affect the bending angle. For PDMS thinner than 30 µm, the PPy layer thickness dominates the bending of the actuator. Therefore, for the flexible section, the PDMS and PPy thicknesses were chosen to be under 30 and 5 µm, respectively, to achieve considerable bending. Theoretically, the thicker the rigid section and the thinner the flexible section, better the control over the bending motion. However, practical handling of a PDMS membrane with high aspect ratio patterns (especially with very thin parts) was difficult and often resulted in the breakage or tearing of the membrane during the fabrication process. Therefore, the chosen dimensions (Table 2) were a trade-off between practical handling and maximum theoretical bending or theoretical rigidity.

**Table 1:** Material parameters used for the simulation of the bending angle of the unimorph microactuator^51–54^

Model parameters	PDMS	Au	PPy
Young’s modulus (MPa)	2.60	80000	3000
Poisson’s ratio	0.50	0.44	0.42
Thickness (µm)	0–200	0.075	1–30
Length of actuator (µm)	500		
Width of actuator (µm)	500		
Relative strain (PPy)	2%

**Table 2:** Design parameters for fabrication of the unimorph microactuator with morphological computation: (A) for the first concept of controlling the bending direction and shape, and B for the second concept of controlling the bending angle

Design parameters	A	B
Thickness of flexible PDMS—Tf (µm)	20	25
Thickness of rigid PDMS—Tr (µm)	100	170
Width of flexible PDMS—Wf (µm)	100	80
Width of rigid PDMS—Wr (µm)	100	500
Thickness of polypyrrole (µm)	5	5

**Fig. 5:**
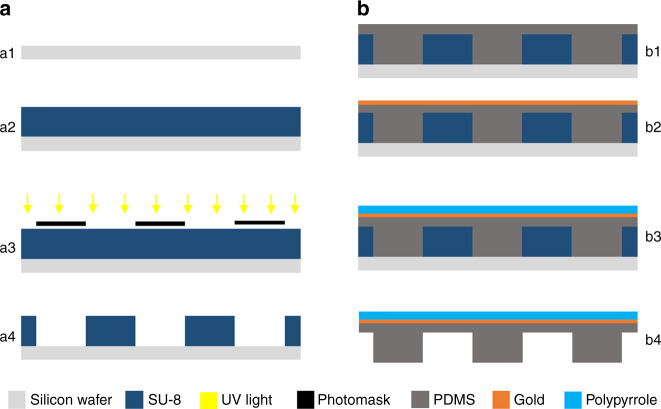
Schematic of the process flow. **a** (a1) Clean silicon wafer as substrate, (a2) spin coat photoresist SU-8, (a3) UV exposure through a photomask, and (a4) dissolve uncured photoresist to develop design template. **b** (b1) Pour PDMS prepolymer and cure under sandwich configuration, (b2) evaporate a thin layer of gold, (b3) electropolymerize pyrrole to form a thick layer of polypyrrole, and (b4) careful release of the PDMS layer from silicon substrate to obtain the unimorph actuator membrane

**Fig. 6:**
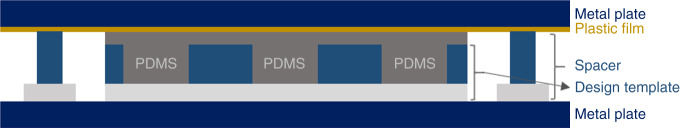
Sandwich configuration to fabricate PDMS membrane with controlled thickness. The difference between the thickness (height) of spacer and design template ultimately defines the thickness of the flexible sections in the PDMS membrane

The actuators were successfully fabricated as explained in the process flow (Figs. 5 and 6) and thereafter released and mounted on a glass slide. The design template could be reused repeatedly, patterning more PDMS membranes/actuators, thus allowing easy manufacturing. It was observed that PDMS membrane being thin and fragile could break during removal from the substrate. The issue was reduced by depositing the gold and PPy layers on top of PDMS before removal, which provided enhanced stability to the membrane. Low adhesion between the cured PDMS membrane and design template is crucial to a successful release of the actuator membrane. The patterned unimorph membrane was then manually cut using a blade under an optical microscope in the shapes as illustrated in Fig. 2.

**Fig. 7:**
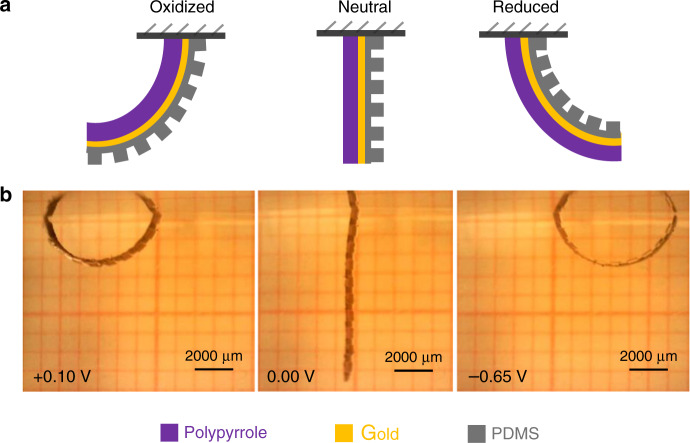
Electrical actuation of the unimorph polymer microactuator. **a** Schematic representation of the orientation of different layers in the microactuator. **b** Bending motion of the microactuator undergoing a redox state change

**Fig. 8:**
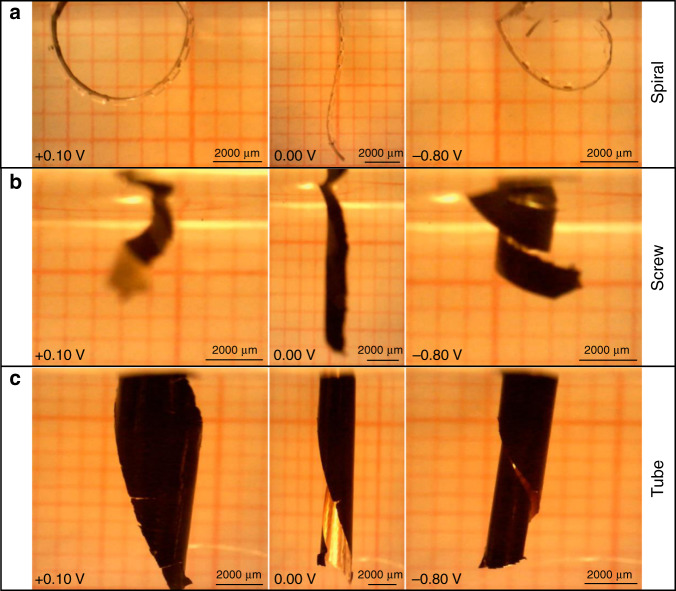
Different bending motions achieved by cutting the same PDMS membrane at different angles with respect to the pattern lines. **a** The motion with perpendicular cut, **b** the motion with slanting cut, and **c** the motion with parallel cut

First, we investigated the feasibility of our process, to see if the actuators operated as intended. Keeping in mind the constraints of handling a fragile PDMS membrane, the parameters for the patterns were chosen to quantitatively control the bending motion, which is to create flexible sections (thinner) that bend and the rigid sections (thicker) that remain flat. We chose the geometrical parameters as listed in Table 2a. Figure 7 shows the actuation of the PDMS-based PPy microactuators upon application of a potential. The fabricated unimorph actuator showed the usual low switching potentials of +0.10 and −0.65 V for oxidation and reduction, respectively, with a fast actuation (2–3 s) between the two states. It can be observed from the figure that the microactuator in reduced state moved through the surface of water and required a bit higher potential and time to overcome the surface tension pull of the water surface. One can see that the bending in reduced state has a smaller radius of curvature and is not limited by the locking mechanism of the rigid sections. To overcome this effect, we applied higher reducing potential (−0.80 V) to get a full bending while exploiting the morphological patterns to guide the motion (Fig. 8a).

### Control of the bending direction and shape

Next, we investigated the first concept of morphological computation by controlling the bending direction and shape. The same unimorph membrane (Table 2a) was cut at different angles to investigate the effect of the angle of the rigid elements on the bending motion. The membrane was cut perpendicular, slanting (~45°) and parallel to the PDMS line patterns, to obtain spiral, screw, and tube kind of bending motions, respectively, as illustrated in Fig. 2. Figure 8a shows the actuator with the line pattern perpendicular to the main axis, bending from the oxidized state (PPy inwards, pattern outwards) towards the reduced state (PPy outwards, pattern inwards). As expected, the actuator bends in a spiral direction. Figure 8b shows the actuator with the patterns at a slanting angle, which moves in an oblique direction to form a screw. Figure 8c shows the actuator with parallel line patterns, folding from the sides to form a tube kind of structure. Unfortunately, due to the clamping of the alligator clip and the length of actuator membrane, it was difficult to achieve a perfect tubular shape. The rigid sections in the actuator are not absolutely rigid and suffer some bending in the direction along the length of the rigid sections on application of potential. The deflection due to bending is more pronounced over large lengths, as in the case of the relatively long tubular structure (length 1000 mm), where we could observe slight bending in the actuator at the far end of the tube length, which resulted in the distorted tubular shape. This was expected from the simulations (Fig. 4) as we chose the rigid parts to be 100 µm thick for practical fabrication reasons, which correspond to bending of 7°. Adjusting the design parameters to accommodate for even thicker rigid parts would alleviate this effect. Nonetheless, this shows that the concept of fabricating soft microactuators with morphological computing using an easy patterning process of soft lithography is feasible.

### Control of the bending angle

Next, we explored the second concept to investigate whether we could have even more precise control of the bending motion with regards to the bending angle using morphological computation. To test the fundamental design theory presented in Fig. 3, we characterized the bending angle of the fabricated actuator in accordance with the described geometric relation. For perfect control over the bending angle, the rigid sections of the microactuator should touch over the corners (locked) to limit the bending motion as illustrated in Fig. 3a. On the contrary, when the thickness or spacing of rigid and flexible sections is not optimum to achieve locking, the bending angle is not controlled by the geometrical locking of the microactuator patterns, but rather determined by only the parameters of various layers of the flexible segment (Fig. 3c). To achieve an optimal locking mechanism for morphological control over bending angle, we designed a spiral unimorph actuator with the parameters as listed in Table 2b. The expected bending angle under a locked condition could be calculated from the simplified equation as:

2 $$\begin{array}{l}{\mathrm{Wf}} = {\mathrm{Tr}} \ast \pi \times \alpha /180,\\ \alpha = {\mathrm{Wf}} \times 180/(Tr \times \pi ),\\ \alpha = 27^\circ. \end{array}$$

**Fig. 9:**
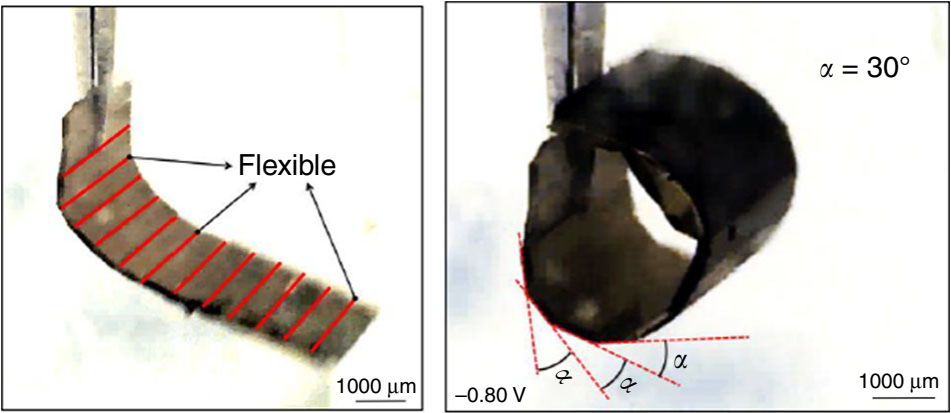
A spiral actuator fabricated and actuated to measure the bending angle for precise control over motion

Figure 9 shows the bending of the microactuator, and as intended, the bending was restricted by the rigid elements touching each other. The rigid sections of the unimorph actuator can be seen locked adjacent to each other, controlling the motion or the extent of bending effectively. The bending angle measured by guidelines over the actuation image is 30°, which is close to the theoretical result of 27° using a simplified geometrical calculation and the deviation is <10%. The inherent bending angle without the patterned PDMS layer restricting the bending should have been 60°, which could be observed from Fig. 4 based on the parameters mentioned above.

**Fig. 10:**
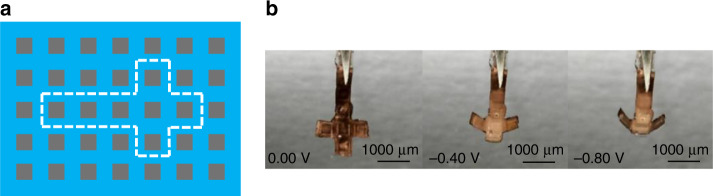
Unimorph polymer actuator with square pattern. **a** Indication of the cut in the square block-patterned unimorph actuator. **b** Electrical actuation of the cut actuator membrane in aqueous ionic solution to form a cube-like structure

Having demonstrated the feasibility of the concept presented in this work (Fig. 8) using the linear pattern, we could even generalize this concept to a square block pattern to form a self-folding cube analogue as demonstrated using conventional microfabrication by Smela et al.^2^. We fabricated a unimorph actuator with squared patterns of PDMS and then cut the membrane as shown in Fig. 10a. Figure 10b shows that the structure starts to fold into a cube-like shape as anticipated. Unfortunately, the assembly of the cube could not perfectly close since the individual layers were not optimized for the complete bending and locking of the PDMS blocks. Further work and optimization are going on to materialize this concept over other various kinds of geometrical structures.

## Conclusion

The developed fabrication process presents a simple method to fabricate multiple microactuator devices from a single design template using soft lithography. The design template was successfully reused to repeatedly pattern PDMS membranes for the fabrication of microactuators. The desired shape and design of the microactuator was decided in the final step of fabrication, making the fabrication easier, with no need for separate or new masks in case of a lateral design change. Patterning the actuators using a geometrical pattern allowed for morphological control over the bending motion. A similar concept of adding rigid elements to flexible bending actuators was done by Smela and Jager using photolithography. They added plates of stiff polymers such as BCB^2^ and SU-8^55^. However, this was not used to control the movement of the microactuators per se, but rather to lift plates that were used as lids or as rigid elements in a microrobot. By using soft lithography, patterning microactuators become simpler since the patterned PDMS layer is directly integrated into the device requiring no extra steps or materials for the morphological computing elements.

We successfully demonstrated the concept of morphological computing in soft microactuators to control the bending motion in two different ways. First, by controlling the bending direction and shape by choosing the pattern direction (cut) with respect to the flexible and rigid elements. Second, by controlling the bending angle (extent of bending) by designing the thickness and spacing of flexible and rigid sections, to enable locking of the rigid elements driven by the flexible sections functioning as joints and hinges.

The adhesion between PDMS and the design template is a limiting factor and needs to be further reduced to fabricate even thinner membranes without mechanical breakage. To be able to cut such thin structures, we will switch to laser ablation to cut out the microactuators, which will also enable smaller structures with finer edges. The thickness of PDMS and PPy plays a crucial role in the actuation and would require further optimization to allow for the fabrication of complex actuator designs such as cubes and cylinders. The presented work utilizes unimorph actuators that work only in ionic solutions, typically aqueous solutions. To enable operation in air, we will construct bimorph structures with an embedded electrolyte layer as a sandwich between two active conductive polymer layers^41,56^. We will use ionogels instead of PDMS for preparing membranes using soft lithography and deposit PPy on both sides to fabricate in-air morphologically computing actuators^57,58^. Such ionogels have high ionic conductivity resulting in fast, in-air actuation and can be micropatterned with morphological geometries using soft lithography. The concept can be generalized into more generic design templates comprising of complex integrated structures, from which a plethora of smart actuators can be made with embedded morphological computation.

## Materials and methods

### Materials

Negative photoresist SU-8 3035 and the corresponding developer mr-dev 600 were acquired from Microchem and used as received. PDMS in the form of Sylgard 184 (base and crosslinking curing agent) was obtained from Dow Corning and was mixed in the ratio 10:1 before use. Pyrrole was purchased from Sigma-Aldrich and was distilled and stored at −20 °C before use. Sodium dodecylbenzenesulfonate (NaDBS) and dichlorodimethylsilane were acquired from Sigma-Aldrich and used as received. As a substrate for the SU-8 design template in photolithography, standard 4-in. silicon wafers were used. The reference electrode (model RE-5B) used for electropolymerization and electrical characterizations was purchased from BASi.

### Template design and fabrication

The geometrical patterning of the PDMS membrane starts with designing of the photomask, which was done with the help of cad software Layout Editor (version 20170115) from juspertor GmbH. The printed photomasks were obtained from JD Photo Data, UK. The design template was fabricated by spin coating (500 r.p.m., 150 r.p.m./s, 10 s + 1000 r.p.m., 150 r.p.m./s, 45 s) and conventional photolithography of the photoresist SU-8 3035 (80 µm) on silicon wafer. The spacers are then fabricated by spin coating (500 r.p.m., 150 r.p.m./s, 10 s + 1000 r.p.m., 150 r.p.m./s, 60 s) and photo crosslinking of SU-8 3035 (100 µm) over another silicon wafer.

### PDMS membrane preparation

Conventional spin coating deposits an inadequately uniform layer of PDMS over the design template, mainly due to the high aspect ratio of the micropatterns. Therefore, to fabricate thin patterned membrane of PDMS, we employed a sandwich type of fabrication method (Fig. 6)^59^. First, the design template is silanized using dichlorodimethylsilane, along with a plastic color copier film to prevent sticking of the fragile PDMS membrane. Then, PDMS prepolymer is mixed, degassed in vacuum and poured over the design template. Silanized plastic film is then placed on top to allow easy removal after curing. This, along with the spacers on the corners, is sandwiched between two flat metal (or glass) plates using clamps to apply pressure. The setup is then placed in an oven at 70 °C and cured for 60 min. Thereafter, the assembly is opened after cooling down at room temperature for 30 min, and the plastic film is carefully removed with a gentle rolling motion from one corner towards the opposite one.

### Preparing conducting polymer microactuators

Next, the PDMS membrane is covered with a thin layer of gold (75 nm) using thermal vacuum evaporation to make it conductive. Then, a thick PPy layer is synthesized using electropolymerization in an aqueous ionic solution of 0.1 M NaDBS containing 0.1 M pyrrole monomers. Electropolymerization was done in a three-electrode (working, counter, and reference) electrochemical cell using a potentiostat (Compactstat, Ivium) to apply an oxidizing potential of 0.6 V (versus Ag/AgCl) for 20 min at room temperature. The PDMS membrane with conducting polymer layers is then cut from the borders and carefully removed from the design template to obtain the actuator membrane as shown in Fig. 2. The final shape of the microactuators with the morphological computing pattern is then cut using a blade.

### Electrical actuation

Electrical actuation was done in an aqueous solution of 0.1 M NaDBS. The setup consisted of an electrochemical cell with three electrodes (working, counter, and reference) and potentiostat (Compactstat, Ivium), along with a portable USB microscope (Edge, Dino-Lite) to record the movement of actuators. All the electrical potentials were measured versus Ag/AgCl as reference electrode. The distance between the microscope and actuator was carefully adjusted to match the monofocal lens of the microscope to try and keep the entire actuator in focus during actuation motion. To ensure enough exposure for brighter images, an external lamp was introduced as well.
